# What Is the Best High Potency

**Published:** 1893-02

**Authors:** 


### HOMŒOPATHIC PHYSICIAN,

A MONTHLY JOURNAL OF

HOMŒOPATHIC MATERIA MEDICA AND CLINICAL MEDICINE.

"If our school ever gives up the strict inductive method of Hahnemann, we are lost, and deserve only to be mentioned as a caricature in the history of medicine."—CONSTANTINE HERING.

Vol. XIII. FEBRUARY, 1893. No. 2.

### EDITORIAL.

WHAT IS THE BEST HIGH POTENCY to use by a physician who wishes to change his practice to high potencies? is a question frequently addressed to the editor of this journal.

To all such, an answer is herewith given that the laborious writing of personal letters to individual inquirers may cease.

The answer may be thus stated: *no* potency can be considered the *best*. No physician can abruptly change his method of practice from low potencies to high.

Judgment in selecting potencies must come from industrious pains-taking effort on the part of the physician seeking to cure his patient in the shortest time and by the "safest, and most certain means upon principles that are at once plain and intelligible."

The using of high potencies does not necessarily make a physician a good homœopathist, though there are many who seem to think so, consequently there is a prevalent impression that this question of potency has divided the homœopathic school into two factions.

It is doubtful if this be the real cause of the existence of the two factions, because the uncertainties of the potency question, the varying experience of different observers, produce such a conflict of testimony as to preclude the formation of any partisan lines.

The real cause of the division would seem to be that there are two orders of mind, one of which seeks only the simillimum and is content to administer it alone, whilst the other one neglects to make any serious effort in this direction, but supplements its deficiencies in this respect by resorting to the methods of the old school, which are therefore classed under the name of "adjuvants." The division upon these grounds is not usually acknowledged by the latter order, but they charge it to a difference of opinion upon the question of potency. Thus this "prevalent impression," before spoken of, gains ground with the uninitiated.

It is noticeable, with those who use this mixed treatment, that the "adjuvants" command the larger share of their attention in making prescriptions, while the selecting of the simillimum plays only a secondary part. Some even claim, when exception is taken to their prescribing massive physiological doses of Quinine, and other drugs as homœopathic physicians, that it is only a raising of the potency question.

Now all these ideas must be eradicated from the minds of those who would realize the deep satisfaction of bringing about a cure by homœopathic prescribing.

They must set aside all thought of using high potencies until they have become proficient in selecting the similar remedy. They must earnestly seek the simillimum in every case and prescribe it. They must watch for the results and if they have been correct in their selection they will get such responses from nature as will convince them, without the need of any one's assertion or persuasion that *Similia similibus curantur* is a universal law. They will realize that it is of *less* importance whether the dose given be a low or a high potency, but it is of supreme importance that the remedy chosen be the simillimum to the disease; or, to speak more accurately, the *sick condition.*

The relative significance of these two terms "disease" and "sick condition" as used in the foregoing lines will become more apparent to them. Familiarity with the materia medica will be obtained and a higher ability to examine the patient to find the "guiding symptoms" will be acquired. The conditions under which a low potency will prove unsatisfactory and need to be supplemented by the higher, will be apparent, and thus step by step a fund of experience upon the best potencies to use will accumulate and so an intelligent view of the potency question afforded.

If it seem that too much is here promised, the only reply that can be made is, *try* the experiment. Seek the simillimum and abandon all means else, of treatment, and it will be found that this higher knowledge can be acquired. It cannot be obtained by adopting the teachings of another who may have been through the discipline. It does not come as a sudden conviction. It is rather a slow growth of observation and meditation gradually ripening into experience and deep conviction, that no opposition can repress, no ridicule weaken; but which arms its possessor with a courage and hope that nerves him to stand and calmly await the results of the simillimum in the most desperate cases.

